# Comprehensive Analysis of Dementia Types and Risk Factors: A Study From a Tertiary Care Center in India

**DOI:** 10.7759/cureus.62745

**Published:** 2024-06-20

**Authors:** Mayank Mundada, Pradnya M Diggikar, Ankit Shokeen, Raju Hansini Reddy, Arun B Oommen, Tushar Pancholi, Bhavya Yammanuru, Sree vidya Yekkaluru, Janani R, Akhilesh Jagirdar

**Affiliations:** 1 Internal Medicine, Dr. D. Y. Patil Medical College, Hospital and Research Centre, Dr. D. Y. Patil Vidyapeeth (Deemed to be University) Pune, Pune, IND

**Keywords:** neurodegenerative disorders, alzheimers dementia, spectrum of dementia, dementia etiology, risk factors of dementia, reversible dementia

## Abstract

Background and objective

Dementia is a prevalent clinical syndrome characterized by memory impairment and cognitive dysfunction. Its global burden is expected to rise significantly, particularly in low- and middle-income countries. Understanding the spectrum of dementia types and associated risk factors is crucial for effective management. This study aims to elucidate the demographic profiles, clinical types, and risk factors of newly diagnosed dementia cases at a tertiary care hospital in India.

Methods and materials

A cross-sectional, hospital-based observational study was conducted on 81 patients at the Department of Medicine, Dr. D. Y. Patil Medical College, Hospital, and Research Centre, Pimpri, Pune, from February 2022 to January 2024. Ethical approval was obtained, and written consent was obtained from participants. Clinical diagnosis was based on the Diagnostic and Statistical Manual of Mental Disorders, Fifth Edition (DSM-V) criteria, supported by cognitive assessment tools and laboratory/radiological investigations. Inclusion criteria encompassed individuals aged 18 years or older, presenting with clinical symptoms suggestive of dementia, having a Mini-Mental State Examination (MMSE) score of less than 24 and Montreal Cognitive Assessment (MoCA) score of less than 25, according to DSM-V criteria for dementia. Exclusion criteria included individuals with a history of head trauma or those below 18 years of age.

Results

Of the 81 participants, the majority (74.1%) were over 60 years old, with females comprising 59.3% of the sample. Alzheimer's disease was the most prevalent dementia subtype (34.5%), followed by vascular dementia (19.7%) and mixed dementia (13.5%). Other causes included Lewy body dementia (2.46%), Parkinson's dementia (4.9%), frontotemporal dementia (4.9%), and Creutzfeldt-Jakob disease (1.2%). Reversible causes accounted for a significant proportion of cases: alcohol-associated dementia (6.1%), hypothyroid-associated dementia (3.7%), HIV-associated dementia (2.46%), herpes simplex dementia (1.2%), neurosyphilis-associated dementia (1.2%), and normal pressure hydrocephalus (NPH)-associated dementia (2.4%). Analysis of risk factors revealed distinct patterns among different dementia types, emphasizing the role of cardiovascular and metabolic health.

Conclusion

This study provides insights into the demographic profiles, clinical types, and dementia risk factors in India. Addressing causes and managing cardiovascular/metabolic health is crucial for dementia prevention and management. Comprehensive care strategies and ongoing research efforts are essential for improving dementia outcomes and enhancing the quality of life for affected individuals and their families.

## Introduction

Dementia is a clinical syndrome marked by memory impairment, disruptions in daily activities, altered behavior and personality, and various other cognitive dysfunctions [1]. Globally, approximately 50 million individuals are currently living with dementia, a number expected to soar to 152 million by 2050. This increase is especially pronounced in low- and middle-income countries, which are home to about two-thirds of the global dementia population [2]. While a meta-analysis and systematic review have concentrated on the prevalence of young-onset dementia (YOD), this study identified an age-standardized prevalence of 119.0 per 100,000 population. However, data on the prevalence in low-income countries and younger age ranges remain limited [3]. Dementia can be divided into two main categories: primary (degenerative) and secondary (acquired). The most prevalent degenerative dementias include Alzheimer's dementia, frontotemporal dementia, Parkinson's associated dementia, and Lewy body dementia. Secondary causes primarily encompass vascular issues, central nervous system infections, trauma, metabolic disturbances, and other reversible or treatable conditions [1]. A wide array of conditions can cause cognitive impairment or dementia, many of which are at least partially reversible. These include metabolic, infectious, toxic, autoimmune, epileptic, psychiatric, structural, and other causes. The American Academy of Neurology recommends that all patients with dementia be screened for hypothyroidism, vitamin B12 deficiency, and structural abnormalities as part of the routine evaluation [4]. Previous studies have shown a link between several modifiable risk factors and a higher prevalence of dementia, particularly in developing countries. These risk factors include lower literacy rates, nutritional status, and metabolic and cardiovascular issues. Additionally, these studies indicated that addressing these risk factors could reduce the risk of cognitive decline and potentially lower the risk of dementia [2,5,6].

Aims and objectives

Our study aims to illuminate the demographic profiles, clinical types of dementia, and risk factors of newly diagnosed dementia cases at a tertiary care hospital in India.

## Materials and methods

Study design and setting

The present study was a cross-sectional, hospital-based observational study conducted at the Department of Medicine, Dr. D. Y. Patil Medical College, Hospital, and Research Centre, Pimpri, Pune. The study period extended from February 2022 to January 2024. Before the commencement of the investigation, approval was obtained from the Institute’s Scientific and Ethics Committee (ethical committee clearance number: IESC/PGS/2022/13). Written consent forms were provided to participants in their languages, ensuring they understood the study’s goals, procedures, and potential risks.

Inclusion and exclusion criteria

The inclusion criteria for the study comprised individuals aged 18 years or older with clinical symptoms suggestive of dementia, a Mini-Mental State Examination (MMSE) score of less than 24, and a Montreal Cognitive Assessment (MoCA) score of less than 25, as per the Diagnostic and Statistical Manual of Mental Disorders, Fifth Edition (DSM-V) criteria for dementia. Individuals with a history of head trauma or those below the age of 18 years were excluded from the study.

Sample size

The sample size was calculated considering the proportion of Alzheimer's patients among patients with dementia as 30%, based on the study "Clinical Spectrum, Risk Factors, and Behavioral Abnormalities among Dementia Subtypes in a North Indian Population: A Hospital-Based Study" by Kushwaha et al. [1]. With a confidence interval of 95% and 80% power, the minimum sample size calculated was 81. The software used was WinPepi version 11.38.

Data collection and consent

A detailed clinical history was taken from all patients, focusing on symptoms of dementia. Patients were examined for signs and symptoms of dementia and diagnosed both clinically and through blood and radiological tests. Clinical diagnosis was based on signs and symptoms, MMSE, and MoCA scores. Blood investigations included a complete blood picture, serum electrolytes, renal function tests, liver function tests, blood glucose level, serum proteins, vitamin B12 and folic acid levels, HIV testing, venereal disease research laboratory (VDRL) test, thyroid profile, vitamin B1 levels, anti-nuclear antibodies (ANA) by IF (immunofluorescence)/BLOT (if indicated), cerebrospinal fluid analysis (if indicated), autoimmune encephalitis panel (if indicated), and electroencephalogram (if indicated). Radiological diagnosis involves computerized tomography (CT) or magnetic resonance imaging (MRI), with contrast as indicated.

Ethical approval and informed consent processes were rigorously followed to ensure compliance with institutional and international standards. The collected data were meticulously analyzed to explore the spectrum of clinical types of dementia in the tertiary care setting.

Statistical analysis

In our study, categorical variables were presented as frequency and percentages. As this is a descriptive study, tests of statistical significance and hypothesis testing were not considered. Data tracking was performed using Microsoft Excel (Microsoft Corporation Redmond, Washington, USA) and analyzed using Jamovi version 2.4.12.0.

## Results

**Figure 1:**
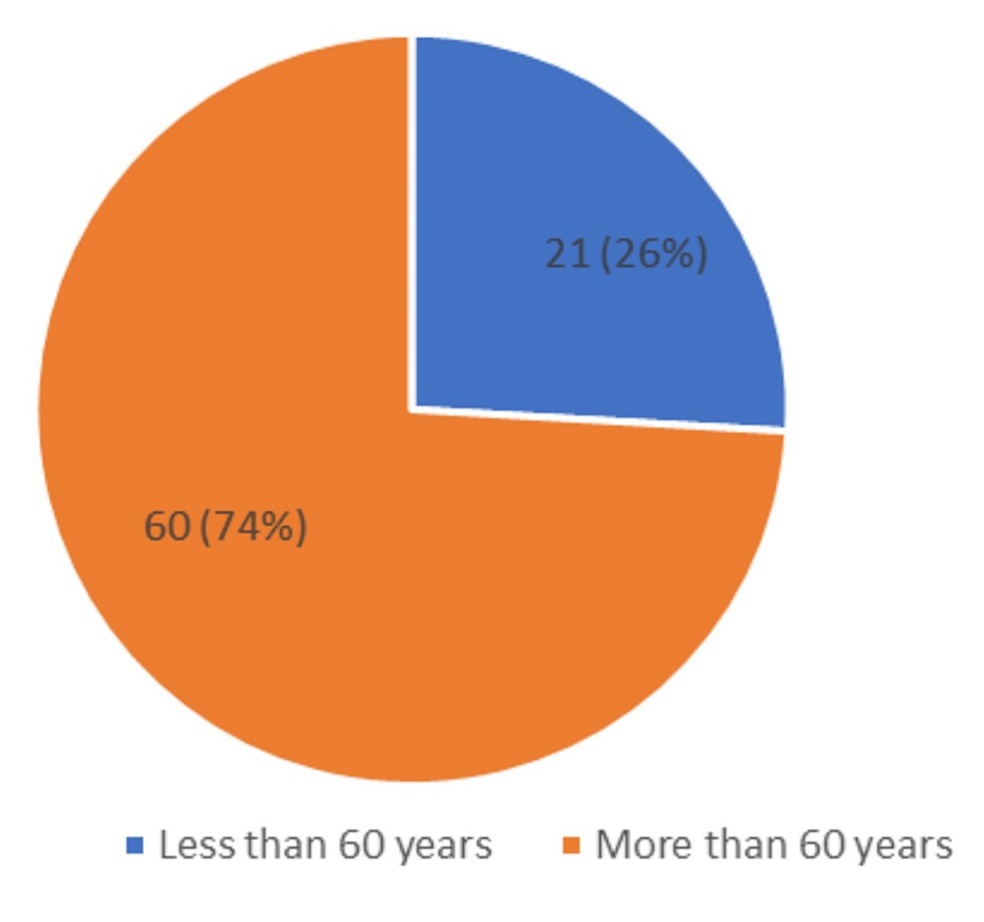
Pie chart showing the age distribution of the study population

**Figure 2:**
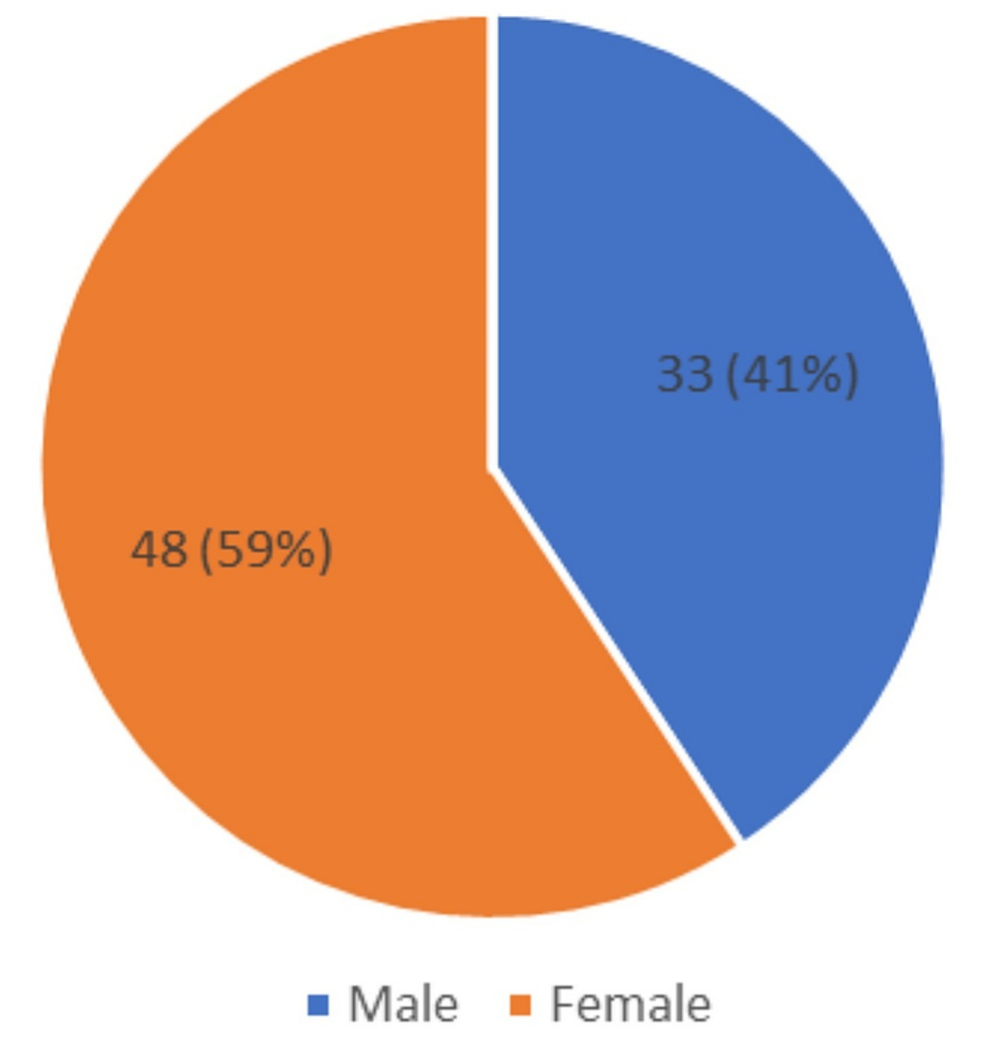
Pie chart showing the gender distribution of the study population

In our study, out of 81 participants, 60 (74%) patients were in the age group of >60 years, while 21 (26%) of them were below 60 years of age (Figure 1). The gender distribution in our study was as follows: 48 (59%) were females, while 33 (41%) were males (Figure 2). This indicates a higher prevalence of dementia in females and older age groups.

Our study analyzed the prevalence of various risk factors among patients diagnosed with different types of dementia: Alzheimer's disease, frontotemporal dementia, mixed dementia, and vascular dementia. The findings are summarized below (Table 1).

**Table 1:** The distribution of risk factors in different subtypes of dementia n = frequency

Risk factors	Alzheimer's disease (n = 28)		Frontotemporal Dementia (n = 4)		Mixed dementia (n = 8)		Vascular dementia (n = 16)	
	Frequency	Percent	Frequency	Percent	Frequency	Percent	Frequency	Percent
Older age	24	85.7	3	75	0	0	4	25
Hypertension	15	53.5	1	25	5	62.5	14	87.5
Diabetes mellitus	5	17.8	1	25	2	25	6	37.5
Ischemic heart disease	2	7.1	0	0	3	37.5	11	68.75
Literacy status	12	42.8	2	50	3	37.5	8	50
Family history of depression/dementia	4	14.2	1	25	1	12.5	2	12.5
Smoking	3	10.7	1	25	3	37.5	2	12.5
Fasting lipid profile	9	32.1	1	25	6	75	11	68.75

The analysis of the data reveals distinct patterns in the prevalence of various risk factors among different types of dementia. In Alzheimer's disease, older age was a significant risk factor, present in 24 patients (85.7%). Hypertension was found in 15 patients (53.5%), diabetes mellitus in five patients (17.8%), ischemic heart disease in two patients (7.1%), literacy status indicating lower education levels in 12 patients (42.8%), a family history of depression or dementia in four patients (14.2%), smoking in three patients (10.7%), and fasting lipid profile abnormalities in nine patients (32.1%).

In frontotemporal dementia, older age was prevalent, accounting for three patients (75%). Hypertension was present in one patient (25%), diabetes mellitus in one patient (25%), literacy status in two patients (50%), a family history of depression or dementia in one patient (25%), smoking in one patient (25%), and fasting lipid profile abnormalities in one patient (25%).

For mixed dementia, older age was not recorded, hypertension was the most common risk factor, present in five patients (62.5%), diabetes mellitus in two patients (25%), ischemic heart disease in three patients (37.5%), literacy status in three patients (37.5%), a family history of depression or dementia in one patient (12.5%), smoking in three patients (37.5%), and fasting lipid profile abnormalities in six patients (75%).

**Figure 3:**
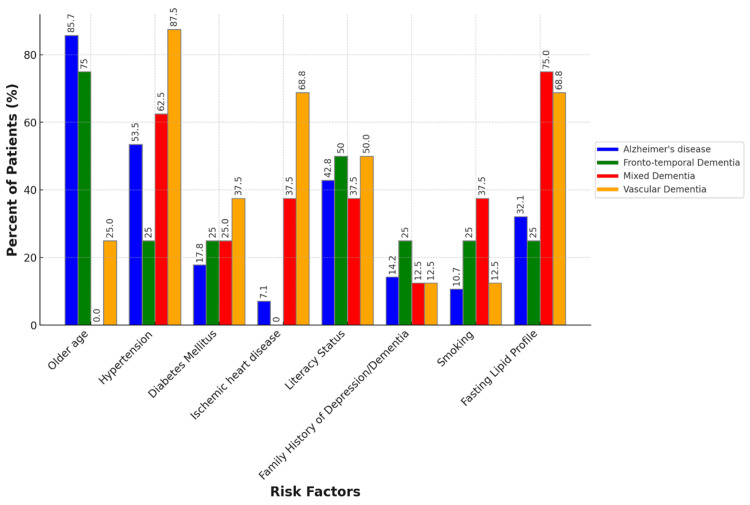
Clustered bar chart showing the distribution of risk factors among different subtypes of dementia

Vascular dementia showed the highest association with hypertension, which was present in 14 patients (87.5%). Older age was a risk factor in four patients (25%), diabetes mellitus in six patients (37.5%), ischemic heart disease in 11 patients (68.75%), literacy status in eight patients (50%), a family history of depression or dementia in two patients (12.5%), smoking in two patients (12.5%), and fasting lipid profile abnormalities in 11 patients (68.75%) (Figure 3). These findings highlight the importance of managing cardiovascular and metabolic health to potentially mitigate the risk of developing certain types of dementia.

**Figure 4:**
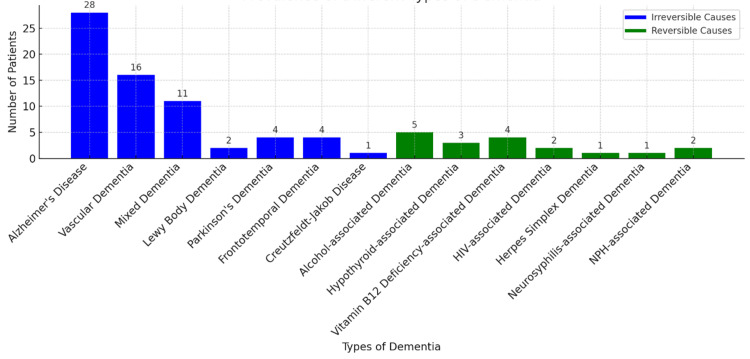
Vertical bar chart showing the prevalence of reversible and irreversible causes of dementia

The study found that the majority of dementia cases were due to irreversible causes. Alzheimer's disease was the most prevalent, affecting 28 patients (34.5%) out of 81. Vascular dementia was the second most common, with 16 patients (19.7%), followed by mixed dementia with 11 patients (13.5%). Other irreversible causes included Lewy body dementia in two patients (2.46%), Parkinson's dementia in four patients (4.9%), frontotemporal dementia in four patients (4.9%), and Creutzfeldt-Jakob disease in one patient (1.2%). Among the reversible causes, the most common was alcohol-associated dementia, affecting five patients (6.1%) out of 100. Hypothyroid-associated dementia was found in three patients (3.7%), and vitamin B12 deficiency-associated dementia was present in four patients (4.9%). Additionally, reversible dementias included HIV-associated dementia in two patients (2.46%), herpes simplex dementia in one patient (1.2%), neurosyphilis-associated dementia in one patient (1.2%), and normal pressure hydrocephalus-associated dementia in two patients (2.4%) (Figure 4). These data underscore the importance of identifying and treating reversible causes of dementia, as they constitute a significant proportion of the cases. Proper diagnosis and management of these reversible conditions can improve patient outcomes and quality of life.

## Discussion

In the present study, most patients were above the age of 60. This finding is comparable to a similar study by Kushwaha et al. [1], which included patients with a median age range from 58 years (for frontotemporal dementia) to 70 years (for Alzheimer's disease). Similarly, Nair et al. [7] featured patients with a mean age of 65.1 years, closely matching our study's age profile. In contrast, Jang et al.'s [8] study from Korea included only patients older than 60 years. Dementia has been strongly associated with increasing age, which is recognized as a major risk factor. In our study, we found that advanced age emerged as a notable risk factor for Alzheimer's disease, impacting the majority of patients with Alzheimer's disease.

In the current study, female patients constituted the majority, aligning with the sex ratio observed in Jang et al.'s [8] study. This contrasts with most previous studies, such as Kushwaha et al. [1], who reported males as the majority across all types of dementia.

Alzheimer's disease emerged as the predominant cause of dementia in the present study, accounting for the majority of cases. This was followed by vascular dementia and mixed dementia. These findings align with those of Kushwaha et al. [1], whose research also identified Alzheimer's disease as the primary subtype of dementia, followed closely by vascular dementia. Alzheimer's disease, followed by vascular dementia, has been recognized as the most common dementia subtype in numerous studies worldwide [9-11].

There are multiple identifiable reversible causes of dementia. These factors, when addressed appropriately, may help reverse or improve cognitive decline in individuals with dementia. Certainly, here's a rearranged version to further reduce similarity: Neurosurgical conditions cover intracranial tumors, normal pressure hydrocephalus, intracranial abscess, and subdural hematoma. Neuroinfections and inflammations involve cerebral vasculitis, meningitis, encephalitis (limbic, herpes, HIV), neurosyphilis, Whipple's disease, sarcoidosis, Lyme disease, metabolic conditions including pituitary dysfunction, hypercalcemia, thyroid disorders (hypo/hyperthyroidism and Hashimoto's encephalitis), parathyroid disorders (hypo/hyperparathyroidism), Cushing's syndrome, hypoglycemia, Addison's disease, vitamin deficiencies (B1, B6, B12, folate), chronic respiratory failure and chronic liver failure. Other conditions consist of limbic encephalitis (neoplastic/autoimmune), depression, epilepsy, substance use (drugs, alcohol), toxins, and sleep apnea [12].

The reversible causes of dementia in our study closely align with the findings of another study conducted by Bello et al. [13]. In our research, alcohol-associated dementia was the most common reversible cause, followed by vitamin B12 deficiency-associated dementia, hypothyroid-associated dementia, and HIV-associated dementia. Bello et al. [13] reported similar etiologies, reinforcing the importance of identifying and treating these reversible conditions.

Recent research from the 2017 Lancet Commission identifies 12 influenceable risk factors for dementia. These factors include low social contact, hypertension, diabetes, less education, hearing impairment, smoking, obesity, depression, physical inactivity, excessive alcohol consumption, traumatic brain injury, and air pollution. Together, they contribute to about 40% of global dementia cases, indicating that targeted interventions could potentially prevent or delay dementia onset [2].

The analysis of our data also reveals distinct patterns in the prevalence of various risk factors among different types of dementia, closely aligning with the findings of Kushwaha et al. [1]. In patients diagnosed with Alzheimer's disease, the most common associated risk factors were older age and hypertension. For frontotemporal dementia, older age, and literacy status were prevalent risk factors. Mixed dementia showed a high association with abnormal fasting lipid profiles and hypertension. Vascular dementia was predominantly associated with hypertension, abnormal fasting lipid profiles, and ischemic heart disease. These findings underscore the critical importance of managing cardiovascular and metabolic health to potentially mitigate the risk of developing certain types of dementia. The similarity of our results to those reported by Kushwaha et al. [1] further validates these risk factors and their impact on dementia, reinforcing the need for targeted interventions in these areas.

In a study of 1,610 participants by Bransby et al. [14], 66.5% reported modifiable dementia risk factors (MDRFs) across multiple domains, including cardiovascular conditions, mood symptomatology, cognitive/social engagement, risky lifestyle behaviors, and sleep disorders. The study found that those with MDRFs in three to five domains had poorer learning, memory, and attention, along with greater cognitive concerns. These findings suggest that MDRFs are linked to cognitive decline, indicating that multidomain behavioral interventions could help prevent cognitive impairment. Our study also yielded similar results, reinforcing these conclusions.

In a study by Canevelli et al. [15] exploring the effects of diet and nutrition on dementia, certain nutritional compounds and regimens were found to provide significant cognitive benefits to older adults in placebo-controlled trials. However, conclusive evidence is still lacking. More randomized controlled trials with extended follow-up periods are necessary to confirm whether specific dietary components or patterns can reduce the risk of cognitive decline and dementia. A review article by Zhao et al. [16] also suggests that nutrition, along with physical activity and sleep, can reduce the risk of dementia and cognitive decline.

With the global increase in dementia cases, there is a growing necessity for robust models for risk prediction. While study authors in high-income countries (HICs) are actively engaged in developing reliable models for predicting dementia risk, the most substantial surge in dementia cases is anticipated in developing countries [17]. Unfortunately, research on dementia in these regions is lacking. Addressing this gap, Stephan and colleagues [18] conducted a study published in The Lancet Global Health. They assessed the applicability of five dementia risk prediction models, originally formulated in HICs, within low- and middle-income countries (LMICs) using data from the 10/66 study. Their observations represent a significant initial stride toward implementing screening initiatives for individuals at high risk of dementia in LMICs.

Limitations

The limitations of our study include its single-center design, which restricts the generalizability of our findings to other settings. As a hospital-based study, there is a potential for selection bias. Additionally, the absence of a comparator group (patients without dementia) prevents us from determining the significance of the association between dementia and potential risk factors. Additionally, the study's cross-sectional design constrains our capacity to establish causal links between risk factors and dementia. Finally, the sample size may not be large enough to detect less common risk factors or types of dementia, potentially limiting the scope of our conclusions.

## Conclusions

In summary, irreversible neurodegenerative diseases like Alzheimer's and other neurodegenerative conditions are major focuses of dementia research. Although incurable, early diagnosis and comprehensive care can improve outcomes. Rare, fully reversible dementia syndromes highlight the need for clinical vigilance. Identifying atypical features should prompt further evaluation beyond standard screening. Even with typical neurodegenerative symptoms, clinicians should investigate reversible factors, as addressing these can significantly improve outcomes. Our study also highlights significant progress in understanding dementia, particularly the risk factors, while recognizing considerable knowledge gaps.

Incorporating healthy habits can enhance neuroplasticity and prevent cognitive decline. Regular exercise, a balanced diet, cognitive stimulation, social engagement, and adequate sleep all support brain health. These practices help strengthen neural connections and improve memory and learning. Promoting these habits is crucial for dementia prevention and overall cognitive well-being.
